# Treatment Failure in a UK Malaria Patient Harboring Genetically Variant *Plasmodium falciparum* From Uganda With Reduced In Vitro Susceptibility to Artemisinin and Lumefantrine

**DOI:** 10.1093/cid/ciad724

**Published:** 2023-11-29

**Authors:** Donelly A van Schalkwyk, Sade Pratt, Debbie Nolder, Lindsay B Stewart, Helen Liddy, Julian Muwanguzi-Karugaba, Khalid B Beshir, Dawn Britten, Emma Victory, Claire Rogers, James Millard, Michael Brown, Laura E Nabarro, Andrew Taylor, Bernadette C Young, Peter L Chiodini, Colin J Sutherland

**Affiliations:** Department of Infection Biology, Faculty of Infectious and Tropical Diseases, London School of Hygiene and Tropical Medicine, London, United Kingdom; Department of Infection Biology, Faculty of Infectious and Tropical Diseases, London School of Hygiene and Tropical Medicine, London, United Kingdom; UK Health Security Agency Malaria Reference Laboratory, Faculty of Infectious and Tropical Diseases, London School of Hygiene and Tropical Medicine, London, United Kingdom; UK Health Security Agency Malaria Reference Laboratory, Faculty of Infectious and Tropical Diseases, London School of Hygiene and Tropical Medicine, London, United Kingdom; UK Health Security Agency Malaria Reference Laboratory, Faculty of Infectious and Tropical Diseases, London School of Hygiene and Tropical Medicine, London, United Kingdom; Department of Infection Biology, Faculty of Infectious and Tropical Diseases, London School of Hygiene and Tropical Medicine, London, United Kingdom; Department of Infection Biology, Faculty of Infectious and Tropical Diseases, London School of Hygiene and Tropical Medicine, London, United Kingdom; UK Health Security Agency Malaria Reference Laboratory, Faculty of Infectious and Tropical Diseases, London School of Hygiene and Tropical Medicine, London, United Kingdom; UK Health Security Agency Malaria Reference Laboratory, Faculty of Infectious and Tropical Diseases, London School of Hygiene and Tropical Medicine, London, United Kingdom; UK Health Security Agency Malaria Reference Laboratory, Faculty of Infectious and Tropical Diseases, London School of Hygiene and Tropical Medicine, London, United Kingdom; Hospital for Tropical Diseases, University College London Hospitals NHS Foundation Trust, London, United Kingdom; Hospital for Tropical Diseases, University College London Hospitals NHS Foundation Trust, London, United Kingdom; Hospital for Tropical Diseases, University College London Hospitals NHS Foundation Trust, London, United Kingdom; Department of Infectious Diseases, Oxford University Hospitals NHS Foundation Trust, Oxford, United Kingdom; Department of Infectious Diseases, Oxford University Hospitals NHS Foundation Trust, Oxford, United Kingdom; UK Health Security Agency Malaria Reference Laboratory, Faculty of Infectious and Tropical Diseases, London School of Hygiene and Tropical Medicine, London, United Kingdom; Department of Infection Biology, Faculty of Infectious and Tropical Diseases, London School of Hygiene and Tropical Medicine, London, United Kingdom; UK Health Security Agency Malaria Reference Laboratory, Faculty of Infectious and Tropical Diseases, London School of Hygiene and Tropical Medicine, London, United Kingdom

**Keywords:** *Plasmodium falciparum*, travelers’ malaria, treatment failure, drug susceptibility, Uganda

## Abstract

**Background:**

Recent cases of clinical failure in malaria patients in the United Kingdom (UK) treated with artemether-lumefantrine have implications for malaria chemotherapy worldwide.

**Methods:**

Parasites were isolated from an index case of confirmed *Plasmodium falciparum* treatment failure after standard treatment, and from comparable travel-acquired UK malaria cases. Drug susceptibility in vitro and genotypes at 6 resistance-associated loci were determined for all parasite isolates and compared with clinical outcomes for each parasite donor.

**Results:**

A traveler, who returned to the UK from Uganda in 2022 with *Plasmodium falciparum* malaria, twice failed treatment with full courses of artemether-lumefantrine. Parasites from the patient exhibited significantly reduced susceptibility to artemisinin (ring-stage survival, 17.3% [95% confidence interval {CI}, 13.6%–21.1%]; *P* < .0001) and lumefantrine (effective concentration preventing 50% of growth = 259.4 nM [95% CI, 130.6–388.2 nM]; *P* = .001). Parasite genotyping identified an allele of *pfk13* encoding both the A675V variant in the Pfk13 propeller domain and a novel L145V nonpropeller variant. In vitro susceptibility testing of 6 other *P. falciparum* lines of Ugandan origin identified reduced susceptibility to artemisinin and lumefantrine in 1 additional line, also from a 2022 treatment failure case. These parasites did not harbor a *pfk13* propeller domain variant but rather the novel nonpropeller variant T349I. Variant alleles of *pfubp1*, *pfap2mu*, and *pfcoronin* were also identified among the 7 parasite lines.

**Conclusions:**

We confirm, in a documented case of artemether-lumefantrine treatment failure imported from Uganda, the presence of *pfk13* mutations encoding L145V and A675V. Parasites with reduced susceptibility to both artemisinin and lumefantrine may be emerging in Uganda.

## Teaser

We identify the *Plasmodium falciparum* A675V K13 gene variant in a case of antimalarial treatment failure and show these parasites exhibit reduced susceptibility to both artemisinin and lumefantrine. Reduced susceptibility to artemisinin and lumefantrine may be emerging concurrently in Uganda.

Clinical management of uncomplicated malaria caused by the parasite *Plasmodium falciparum* utilizes oral drugs combining a rapid-acting artemisinin compound with a partner from a different chemical class. In sub-Saharan Africa, artemisinin combination therapy (ACT) has generally retained high efficacy since implementation from 2004, despite waning efficacy in some Southeast Asian countries characterized by slow parasite clearance after treatment with ACT. This phenotype, which was first reported in 2008 [1, 2], is associated with certain amino acid substitutions in the propeller domain of the *P. falciparum* kelch protein K13 [3, 4]. Although African parasites are known to carry polymorphisms in the *pfk13* gene encoding this protein, these specific variants were not associated with reduced artemisinin susceptibility in vitro or ACT treatment failure in vivo [3, 4]. However, investigations in Rwanda and Uganda described emerging *pfk13* variant genotypes R561H, C469Y, and A675V that are associated with slow postartemisinin clearance in vivo [5, 6] and reduced susceptibility in vitro [7]. Relevant clinical data on treatment outcomes for infections harboring these variants remain limited in scope [8].

Travelers returning from malaria-endemic countries to nonendemic jurisdictions may harbor parasites carrying novel drug resistance-associated genotypes [9, 10] or displaying evidence of treatment failure [11, 12]. We report a case of uncomplicated *P. falciparum* malaria in a United Kingdom (UK) traveler returning from Uganda treated with intravenous (IV) artesunate followed by the ACT artemether-lumefantrine (Riamet; AL). The patient suffered a recrudescence 19 days later that also proved refractory to standard treatment. *Pfk13* genotype and in vitro parasite susceptibility to artemisinin and lumefantrine were investigated and data compared to 6 other *P. falciparum* isolates from travelers to Uganda, to identify parasite factors associated with unsatisfactory clinical outcomes in treated UK malaria patients. Our findings are considered in the wider context of >32 AL treatment failure cases reported to the UK Malaria Reference Laboratory since 2015.

## METHODS

### Clinical Parasite Samples

Blood samples (500–2500 μL) from UK malaria patients, positive for *Plasmodium falciparum* by microscopy, were obtained from the UK Malaria Reference Laboratory (MRL) at the London School of Hygiene and Tropical Medicine (LSHTM). The study was approved by the LSHTM Research Ethics Committee (reference: 14710) and the National Health Service (NHS), London-Chelsea Research Ethics Committee (reference: 18/LO/0738). Written informed consent was given by the individual described as the index case.

### In Vitro Parasite Cultivation

Culture adaptation of clinical isolates was as previously described [13], in complete medium composed of RPMI 1640 (Sigma-Aldrich product R5886) supplemented with 2% (v/v) human AB serum, 5 g/L Albumax II (Thermo Fisher Scientific, 11021045), 10 mM D-glucose, 2 mM L-glutamine, 25 mg/L gentamicin, and 147 μM hypoxanthine. Cultures were maintained at 37°C in sealed flasks under gas (93% N_2_, 4% CO_2_, and 3% O_2_). Cultures were maintained in human A+ blood (NHS Blood and Transplant, UK) with parasitemia <4% and hematocrit of 4%. Laboratory-adapted lines 3D7, Cam3.II^R539T^, and Cam3.II^rev^ [14] were maintained under the same conditions.

### Compounds

Chloroquine diphosphate (product C6628) was purchased from Sigma-Aldrich (Merck). Lumefantrine (product 20678) and dihydroartemisinin (DHA) (product 19846) were purchased from Cayman Chemicals Company. Desethylamodiaquine was a gift from Dr Harparkash Kaur (LSHTM). Other antimalarial drugs were supplied by the Medicines for Malaria Venture, Geneva, Switzerland [15, 16]. Chloroquine stocks were prepared in sterile distilled water. All other compounds were dissolved in dimethyl sulfoxide (DMSO).

### 72-Hour Dose-Response Growth Inhibition Assays

Susceptibility assays for clinical isolates were performed in complete medium, including 2% (v/v) human AB serum, 5 g/L Albumax II, in 96-well microplates in a final volume of 200 μL, as described previously [13]. The plates were incubated at 37 °C in an incubation chamber (Billups-Rothenburg Inc) under culture gas for 72 hours, and stored at −20 °C overnight. Microplates were thawed, samples resuspended and, in a duplicate plate, 100 µL from each well was added to 100 μL of SYBR Green lysis buffer (1:5000 SYBR Green I [Thermo Fisher Scientific, S7563], diluted in 20 mM Tris, 5 mM ethylenediaminetetraacetic acid, 0.008% [w/v] saponin, 0.08% [v/v] Triton X-100, pH 7.5). Fluorescence was read in a Spectramax M3 microplate reader (Molecular Devices) at 490 nm excitation and 520 nm emission and dose-response data evaluated as described [13].

### Ring-Stage Survival Assay

Synchronized ring-stage cultures (0–3 hours postinvasion; 2% hematocrit; 0.5% parasitemia; 1 mL volume) were exposed to 700 nM DHA or equivalent 0.5% (v/v) DMSO for 4 hours from 3 to 7 hours postinvasion in duplicate. Infected cells were washed 4 times in RPMI medium and returned to culture in drug-free complete medium. Survival at 72 hours postinvasion was measured by flow cytometry [17]. In brief, 500 µL of resuspended parasites from each well was transferred to a 1.5-mL tube and centrifuged for 1 minute at 10 000 rpm. The supernatant was removed, and the parasite pellet washed once with 500 µL of Hanks balanced saline solution (HBSS-1A, Capricorn Scientific), resuspended in 500 µL of HBSS containing 0.5 X SYBR green I and 400 nM Mitotracker Deep Red FM (Thermo Fisher Scientific, M22426), and incubated at 37°C for 30 minutes. The dual-fluorophore-labeled parasites were washed twice with HBSS, and fluorescence was detected after excitation at 488 nm and 640 nm in an Attune NxT cytometer (Thermo Fisher Scientific).

### Parasite DNA Isolation

Genomic DNA was extracted from 200 μL pelleted parasite culture using the QIAamp DNA Blood Mini Kit (Qiagen, UK) or the QIASymphony robotic extraction platform as previously described [18], and stored at −20°C.

### Resistance Marker Genotyping

The *Plasmodium falciparum* chloroquine resistance transporter (*pfcrt*) genotype was determined by multiplex quantitative polymerase chain reaction (PCR) and other marker sequences amplified by nested PCR as described [13, 19–21]. Purified amplicons (Supplementary Table 1) were submitted to a commercial provider for sequencing (Genewiz, Bishop’s Stortford, UK).

### Statistical Analysis

*P* values for comparisons were calculated using the Wilcoxon rank-sum test. Estimates of the effective drug concentration preventing 50% of growth compared to DMSO-treated control (EC_50_) were derived from 3 or more biological replicate assays, each with 2 technical replicates. Estimates of parasite survival in the ring-stage survival assay (RSA) were averaged across a minimum of 4 biological replicates.

## RESULTS

### Treatment Failure in UK Falciparum Malaria Cases 2016–2022

**Figure 1.**
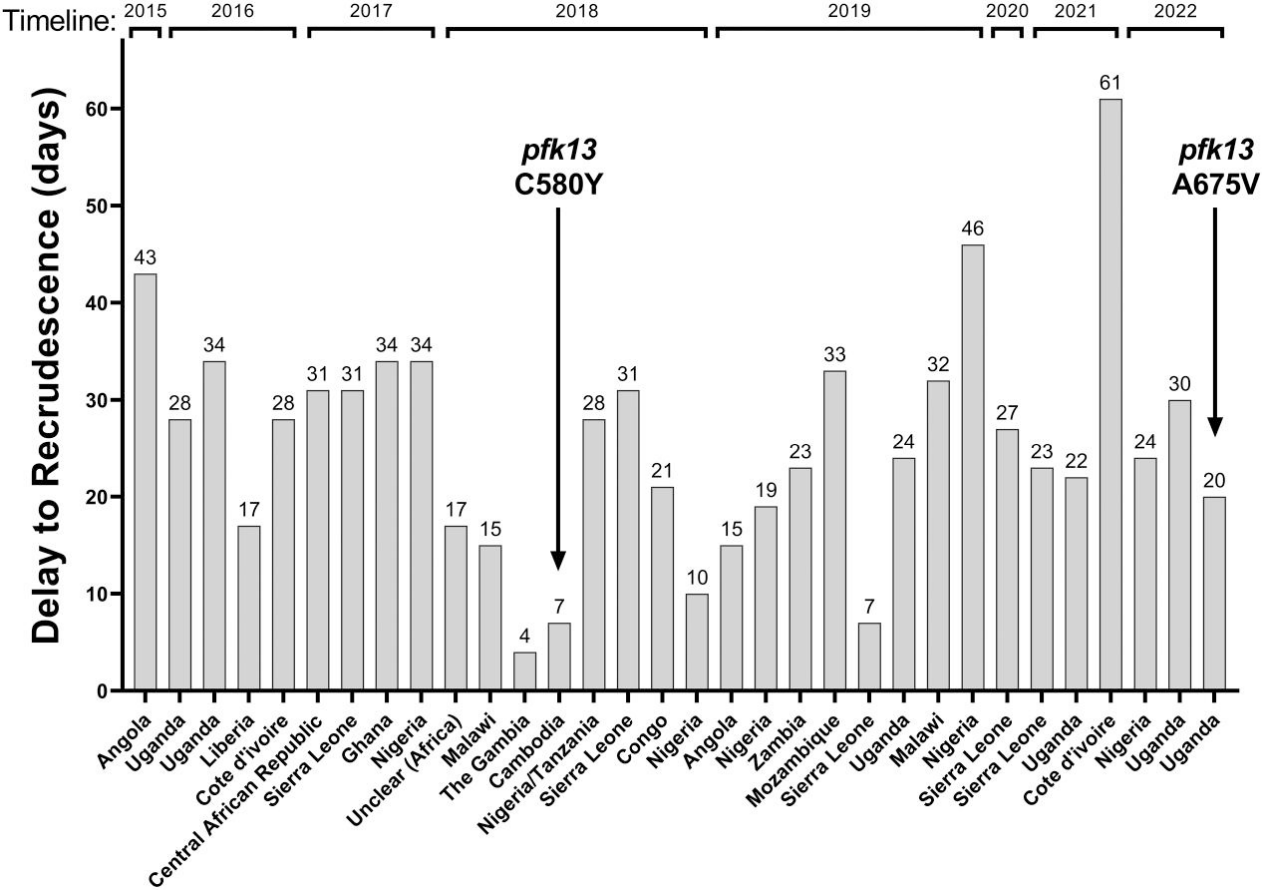
Days to recrudescence in 32 patients in the United Kingdom (UK) with recurrent malaria symptoms and recrudescent parasitemia after artemether-lumefantrine treatment for *Plasmodium falciparum* infection. Cases were identified by surveillance of routine clinic and hospital data for UK malaria patients reported to the UK Malaria Reference Laboratory (MRL) (malaria is “notifiable” in the UK), and by specific recognition of suspected failures by sending hospitals and diagnostic laboratories who alerted the MRL to presentation with malaria symptoms of a previously treated patient. Each bar represents 1 patient; the number given above each bar depicts the time elapsed in days from primary presentation to confirmation of recrudescent parasitemia. Validated K13 propeller domain mutations C580Y and A675V were seen in 1 Cambodian isolate (2018) and the index Ugandan case (2022), respectively. The denominator of total number of reference microscopy-confirmed *P. falciparum* infections referred to the MRL for each year is as follows: 2015: N = 1068; 2016: N = 1308; 2017: N = 1452; 2018: N = 1375; 2019: N = 1475; 2020: N = 437; 2021: N = 868; 2022: data not yet published. Travel restrictions due to the global coronavirus pandemic were in force in the UK in 2020 and 2021.

The UK MRL investigates suspected treatment failure in cases of imported malaria by molecular genotyping of resistance-associated parasite genes [11]. This monitoring relies on voluntary notifications from participating hospitals and laboratories and is not considered exhaustive. Figure 1 presents 32 such cases since 2015, <0.5% of malaria patients notified to the MRL over this 7-year period. Two *P. falciparum* isolates in this series carried mutations in the propeller domain of the *pfk13* locus. The second of these isolates, from 28 October 2022, was from an individual of white British ethnicity without prior history of malaria exposure who had traveled to Uganda and harbored parasites with the A675V variant of *pfk13*. Recent reports from Uganda describe the emergence of this variant since 2021 and provide evidence of association with both slow parasite clearance following treatment in vivo and increased survival in artemisinin susceptibility assays ex vivo. Any role in treatment failure has not as yet been demonstrated [5, 7, 8]. We therefore investigated the October 2022 case in more detail.

### Case History

This 19-year-old man, usually fit and well, traveled to Uganda for 2 weeks in August to September 2022. Sites visited were Murchison Falls, Ishaka, Kampala, and Jinja. Malaria prevention measures included sleeping under mosquito nets, but no chemoprophylaxis was taken. No contacts or other travelers in the party were unwell.

The subject initially became unwell 2 days after returning to the UK (day 2), with fever, lethargy, and a dry cough. He attended hospital and was diagnosed with *P. falciparum* malaria at a parasitemia of 1%, by routine blood film microscopy. No other *Plasmodium* species were present. He was initially tachycardic and hypotensive but improved with IV fluids, with no other features of severe malaria. Laboratory results demonstrated hemoglobin 149 g/L, platelets 212 × 10^9^/L, pH 7.34, and glucose 5 mmol/L. Management was with 3 doses of IV artesunate (2.4 mg/kg) over 24 hours followed by discharge with a course of 6 doses of AL. Although treatment was unobserved, he reported completing the full course of treatment. No clearance blood film was performed after treatment. Post hoc sequencing of the *pfk13* locus indicated the presence of a mixture of both the A675 reference allele and the 675V artemisinin resistance-associated variant.

On day 19, he developed fatigue, cough, headache, and reduced appetite and on day 21 again became febrile. He attended a second hospital on day 23 and was diagnosed with recurrent *P. falciparum* malaria symptoms and a parasitemia of 0.7%. The patient was managed with 2 doses of IV artesunate (2.4 mg/kg) and was transferred to a regional infectious diseases (ID) center on day 25. He received a further dose of IV artesunate (2.4 mg/kg) and was discharged home to complete a second course of AL from day 26–28. He reported taking the full course with full-fat milk to improve absorption as directed, and felt entirely well after completing the course. No clearance blood film was performed after treatment.

On day 51 (25 days after the last dose of AL), he became unwell again with fever, fatigue, and headache; he was admitted to hospital on day 54 and diagnosed with a second recurrence of *P. falciparum* malaria. He was initially hypotensive but had no other features of severe malaria. Bloods showed thrombocytopenia (platelets 64 × 10^9^/L) but were otherwise normal with a normal pH on blood gas. He was treated with 2 doses IV artesunate (2.4 mg/kg) and transferred to the regional ID center. His case was discussed with the MRL given the second recurrence and concern for artesunate partial resistance. Having completed 2 further doses of IV artesunate (2.4 mg/kg), he was switched to oral atovaquone/proguanil (4 × tablets daily) from days 56–58 to complete treatment. The patient remained well following treatment and has had no further recurrences. Repeat blood films on days 61 and 72 were negative for *P. falciparum* trophozoites. Post hoc sequencing of the *pfk13* locus indicated the presence of only the 675V artemisinin resistance-associated variant in the day 19 and day 51 recurrent isolates, consistent with elimination of the susceptible allele.

### Culture Adaptation and Susceptibility Testing of Recrudescent Parasites

**Figure 2.**
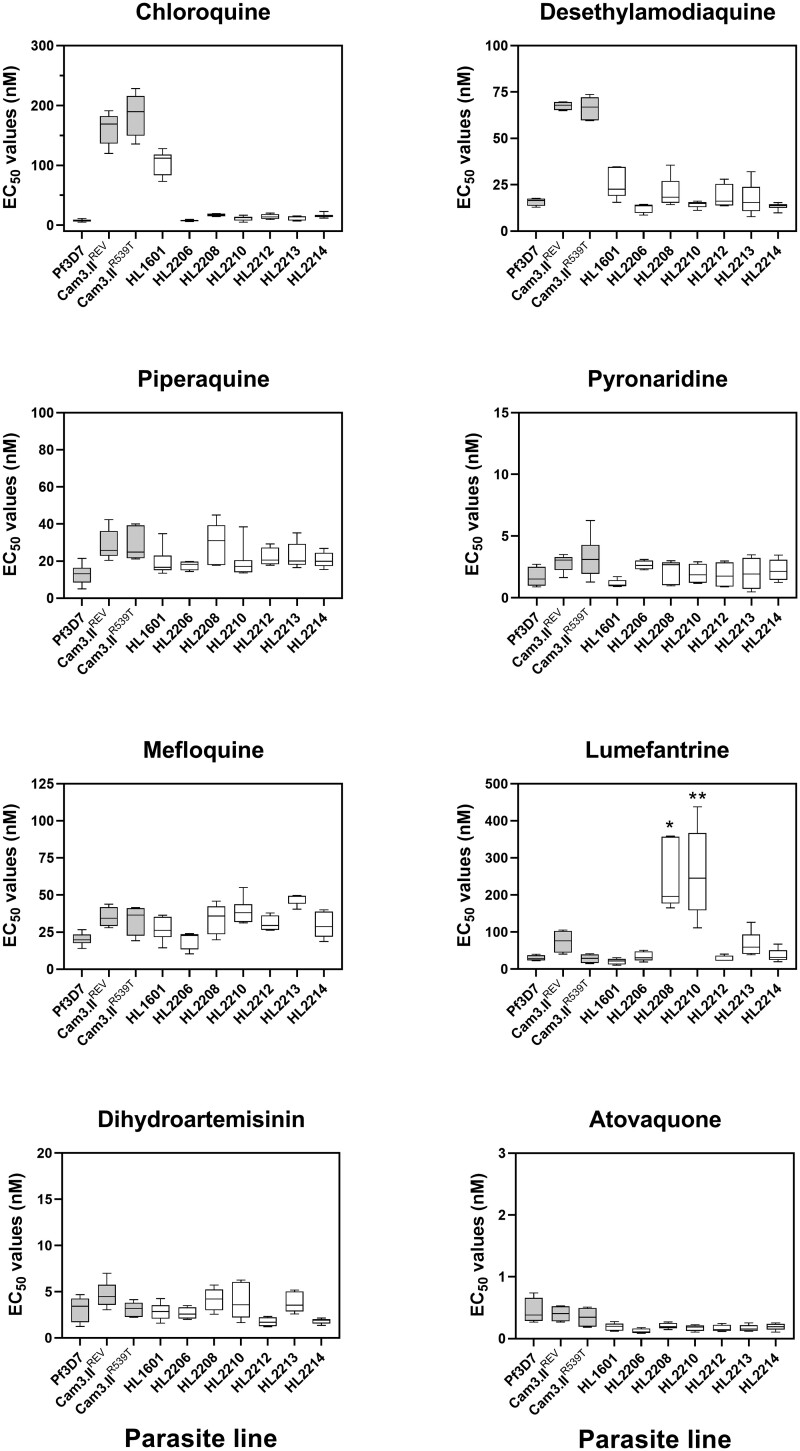
Seventy-two-hour in vitro dose-response assays for 7 Ugandan clinical isolates against 8 antimalarials. Susceptibility is shown as effective concentration preventing 50% of growth (EC_50_) estimates. Each box in the plot indicates the median and interquartile range of 4–8 biological replicates, each performed in duplicate, and whiskers indicate range. Isolate cultures (empty boxes) were not cloned and likely represent a mix of genotypes, which may have contributed to variability among biological replicates of the same isolate. The index case is represented by the line HL2210. *Pairwise Wilcoxon test of lumefantrine EC_50_ compared to 3D7 exact *P* = .016. **Pairwise Wilcoxon test of lumefantrine EC_50_ compared to 3D7 exact *P* = .010.

Recrudescent parasites were isolated from the patient’s third febrile episode, on day 52 since first diagnosis. Blood-stage *P. falciparum* parasites were successfully culture-adapted, expanded in vitro, and cryopreserved with the isolate designation HL2210. This line, together with 6 other uncloned, culture-adapted *P. falciparum* isolates of Ugandan origin (Table 1), were investigated, by standard 72-hour parasite growth inhibition assays, for reduced susceptibility to a panel of 6 compounds recommended for malaria treatment or prophylaxis in Africa and the UK: DHA, lumefantrine, piperaquine, atovaquone, desethylamodiaquine, and chloroquine. Estimates of EC_50_ for each line, for each drug, were compared to estimates obtained for control lines 3D7, Cam3.II^R359T^ (artemisinin-tolerant Cambodian isolate), and Cam3.II^rev^ (artemisinin-susceptible revertant engineered from Cam3.II^R359T^) (Figure 2) [14]. HL2210, the culture-adapted recrudescent parasite line, did not display significantly reduced susceptibility to DHA in the 72-hour exposure assay but displayed a very high EC_50_ for lumefantrine of 259.4 nM (95% confidence interval [CI], 130.6–388.2; n = 6), significantly greater than that for the fully susceptible reference line 3D7 (29.72 nM [95% CI, 17.91–41.53]; n = 4; *P* = .001). HL2210 was fully susceptible to chloroquine and piperaquine. Among the 6 other Ugandan isolates tested, cultured line HL2208 also displayed significantly reduced susceptibility to lumefantrine, with an EC_50_ estimate of 252.7 nM (95% CI, 133.6–371.8; n = 5; *P* = .016 compared to 3D7). All 6 comparator lines were fully susceptible to piperaquine, with HL1601 alone resistant to chloroquine (EC_50_ estimate, 102.6 nM [95% CI, 83.9–121.4]; n = 7).

**Table 1.** HL Series Culture-Adapted *Plasmodium falciparum* Lines From UK Travelers Returning From Uganda

Serial	Line Name	Year Received	Countries Traveled	Treatment Failure	Notes
1	HL1601^a^	2016	Uganda	Yes	MRL series: case 2
2	HL2206	2022	Uganda	No	Suspected HRP deletion
3	HL2208	2022	Uganda	Yes^c^	MRL series: case 31
**4**	**HL2210^b^**	**2022**	**Uganda**	**Yes (**× **2)**	**MRL series: case 32**
5	HL2212	2022	Uganda	No	…
6	HL2213	2022	Uganda	No	…
7	HL2214	2022	Uganda	No	…

Abbreviations: HRP, histidine-rich protein; MRL, Malaria Reference Laboratory.

^a^HL1601 was established from a case previously described in reference [11].

^b^The in vitro cultured line derived from the index case is shown in boldface. Comparator lines were selected by “convenience” as Ugandan traveler blood samples that we were able to establish in culture, and were collected in 2022, the same year as the index case.

^c^Clinical data for this case were limited; the patient was a 24 year-old woman who presented with a positive rapid test and 3.8% *Plasmodium falciparum* parasitemia by microscopy. Treatment was with artemether-lumefantrine, following UK guidelines. No blood sample from this initial episode was received by the MRL. This individual presented with recurrence of symptoms 30 days later, at which time a blood sample was received at the MRL and successfully placed in culture (HL2208).

**Figure 3.**
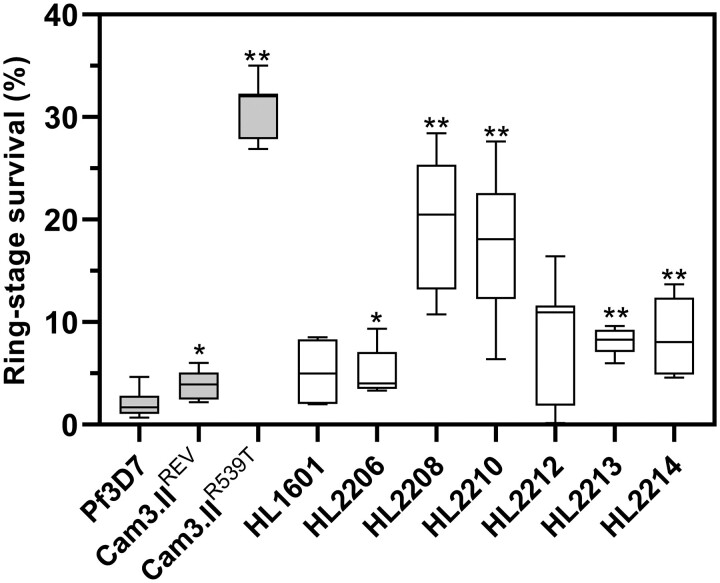
Ring-stage survival assay (RSA) survival estimates for 7 Ugandan clinical isolates, compared to 3D7 and Cambodian comparator lines. Susceptibility is shown as percentage parasite survival (compared to dimethyl sulfoxide control) 72 h following exposure to a 4-h pulse of 700 nM dihydroartemisinin. Box and whiskers indicate median, interquartile interval, and range. UK traveler isolates are shown with empty boxes. Number of interpretable biological repeats (each with 2 technical replicates) from left to right: 13, 10, 8 (control lines), 4, 8, 8, 14, 8, 8, 8 (clinical isolates). The index case is identified by a vertical arrow. *Pairwise Wilcoxon test compared to 3D7 exact *P* < .05. **Pairwise Wilcoxon test compared to 3D7 exact *P* < .001.

Reduced susceptibility to artemisinins as observed in Southeast Asia is assessed by the RSA [3, 14, 22]. Synchronized early ring-stage parasite cultures (0–3 hours post–erythrocyte invasion) are exposed to a 4-hour pulse of 700 nM DHA [23], after which drug is washed off and relative survival compared to DMSO-treated control culture wells after a further 68-hour incubation. In the RSA, HL2210 displayed a mean relative survival of 17.3% (95% CI, 13.6%–21.1%; n = 14) significantly greater than seen for 3D7 (1.99% [95% CI, 1.27%–2.70%]; n = 13; *P* < .0001). Of the 6 comparator lines of Ugandan origin, all displayed relative survival of <10%, similar to fully susceptible controls, except for HL2208, which displayed mean survival in the RSA of 19.8% (95% CI, 14.5%–25.2%; n = 8; *P* = .0002) (Figure 3).

### Genotyping of Parasite Lines From Uganda

The genotype of each Ugandan parasite line was determined at resistance-associated loci *pfk13*, *pfcrt*, *pfmdr1*, *pfcoronin*, *pfap2mu*, and *pfubp1* (Table 2; Supplementary Figures 1 and 2) [20, 21]. Only parasites from our index case (HL2210) carried a *pfk13* propeller domain mutation, the A675V variant as seen in the patient blood sample, together with a novel L145V nonpropeller variant. HL2210 also carried an unusual cysteine residue in *pfubp1*, Y1530C, among the previously described KYD repeats (Supplementary Figure 2) [21]. The significance of this variant is unknown, but the inclusion of a cysteine residue in a tract of low complexity is unexpected. HL2208 carried the uncharacterized T349I *pfk13* nonpropeller variant, the *pfcoronin* P76S gene variant (widespread in Africa but not yet associated with artemisinin susceptibility [25]), and an R3105L variant in *pfubp1* and *pfap2mu* S160N (also moderately common across Africa but of uncertain selective advantage in AL-treated individuals) [21, 24, 26]. Another Ugandan comparator line, HL2213, carried the F437L variant of *pfap2mu* which, although previously described in samples from Kenya [21], has not been associated with any susceptibility phenotype. Variable lengths of asparagine tracts in *pfap2mu* were evident among the isolates tested (Supplementary Figure 1). Variation was also found among isolates in the 3- and 9-amino acid repeat regions of *pfubp1* (Supplementary Figure 2).

**Table 2.** Drug Resistance-Associated Genotypes in *Plasmodium falciparum* Lines From Uganda

Line Name	*Pfk13* Propeller	*Pfk13* Nonpropeller	*Pfcrt* Codons 72–76	*Pfmdr1* Codons 86 and 184	*Pfmdr1* CN	*Pfubp1* 3-aa Repeats^a^	*Pfubp1* 9-aa Repeats^b^	Coronin	*Pfap2μ*
HL1601	Ref	Ref	CVIET	NY (Ref)	1	1,3,1	2,1,0,3	Ref	Ref
HL2206	Ref	Ref	CVMNK (Ref)	NY (Ref)	1	Ref	2,1,0,3	Ref	Ref
HL2208	Ref	T349I	CVMNK	NF	1	Ref	2,1,0,3 R3105L	P76S	S160N
HL2210	A675V	L145V	CVMNK	NF	1	3,1,1 Y1530C	Ref	Ref	Ref
HL2212	Ref	Ref	CVMNK	NF	1	Ref	1,0,1,2	Ref	Ref
HL2213	Ref	Ref	CVMNK	NF	1	Ref	2,1,0,3	Ref	F437L
HL2214	Ref	Ref	CVMNK	NF	1	2,1,1	tbd	Ref	Ref

“Ref” corresponds to reference genome sequence (Pf3D7). For each drug investigated in this paper, 3D7 is considered fully susceptible and therefore “wild type” at these loci.

Abbreviations: aa, amino acid; CN, gene copy number estimate by quantitative polymerase chain reaction as previously described [24]; tbd, to be determined.

^a^Refers to *pfubp1* (PF3D7_0104300) codons 1463–1563 (Supplementary Figure 2); ref format is 2,2,1.

^b^Multiple 9-aa repeats in *pfubp1* codons 3064–3126 (Supplementary Figure 2); ref format is 2,1,0,4.

## DISCUSSION

We describe a UK malaria patient who experienced recrudescent *P. falciparum* parasitemia after 2 separate courses of AL treatment 19 days apart, thus displaying clinical treatment failure, and confirmed the presence of the A675V variant of *pfk13* in peripheral blood samples at all 3 time-points. We then demonstrated that a parasite culture derived directly from the patient’s third malaria episode displayed significant in vitro survival in the RSA. This provides evidence that the A675V *pfk13* variant, known to mediate bona fide artemisinin partial resistance, manifested as slow clearance in vivo [5, 7], can also contribute to AL treatment failure. Equally important, we present evidence of significantly reduced susceptibility to lumefantrine in vitro, suggesting that *P. falciparum* populations in Uganda are under selection by this important partner drug. Worryingly, a contemporary isolate of Ugandan origin also derived from a UK malaria patient with post-AL recrudescence, but not harboring a *pfk13* propeller domain mutation, showed similar reduction in susceptibility to both artemisinin and lumefantrine. These results suggest that mutations in *pfk13* are insufficient on their own to cause clinical failure in AL-treated African patients, but that additional parasite adaptation that reduces susceptibility to lumefantrine is emerging independently. We conclude that reduced susceptibility to lumefantrine may be emerging in Uganda in concert with new *pfk13* variants, posing a potentially serious threat to therapeutic management of falciparum malaria.

Our study has limitations. First, there are no validated genetic markers for lumefantrine susceptibility that could support our findings. AL susceptibility is inversely correlated with susceptibility to chloroquine and amodiaquine, as evidenced by directional selection on the *pfmdr1* and *pfcrt* resistance loci in East Africa from 2005 [21, 27–30]. Those genotypes favored in populations where AL was deployed should not be considered sufficient on their own to cause reduced susceptibility to AL, but more as markers of lumefantrine selection. Second, our elevated lumefantrine EC_50_ estimates for HL2208 and HL2210 in vitro are inconsistent in absolute value with estimates for recent Ugandan isolates reported by others [31], which are known to display wide error estimates (Figure 2). In mitigation, the inclusion of established comparator lines provides internal experimental validity and permits comparison of relative differences in susceptibility with other laboratories. Third, we present a case report only, with unobserved oral dosing and without drug level measurements. This diminishes generalizability of our findings. Finally, further work with these isolates is required to establish the durability of the phenotypes we have measured, as EC_50_ can drift markedly following recovery from cryopreservation [7].

Systematic investigation of the genomic determinants of emerging artemisinin partial resistance is now needed across Africa coupled with prevalence studies that include the recently identified, unvalidated candidates from Uganda, *pfk13* P441L and V469F [32], and the validated *pfk13* marker from Eritrea, R622I [33]. Identification of validated markers for reduced lumefantrine susceptibility are urgently needed for surveillance, so that alternative combination regimens can be implemented where most needed. We noted that HL2210 and HL2208 carried genetic variants of interest in *pfubp1*, *pfap2μ*, and *pfcoronin* as well as in the nonpropeller domains of *pfk13* and that these may be contributing to either or both artemisinin and lumefantrine susceptibility phenotypes, but we cannot yet verify these as reliable surveillance markers. It is our view that falling lumefantrine susceptibility is the greater threat to ACT efficacy right now, so it is a priority to generate evidence that other partner drugs such as pyronaridine, piperaquine, and amodiaquine remain efficacious in alternative ACT regimens. Our in vitro data on the recent Ugandan isolates confirm susceptibility to these partner drugs (Figure 2), but in vivo evidence is also needed. In endemic countries, sequential courses of 2 different ACTs over 6–7 days may be a simple and effective way to manage *P. falciparum* in localities where cases of treatment failure have been confirmed [34]. In the UK and Europe, the recommended and most widely used antimalarial combination, AL, has also been the most widely used ACT across Africa for the past 20 years. Therefore, currently evolving antimalarial resistance in Africa presents a serious threat to effective malaria treatment in Europe. Attention should now turn to ensuring alternative ACTs such as DHA-piperaquine are readily available for treatment of recrudescent East African *P. falciparum* infections.

## Supplementary Data

Supplementary materials are available at *Clinical Infectious Diseases* online. Consisting of data provided by the authors to benefit the reader, the posted materials are not copyedited and are the sole responsibility of the authors, so questions or comments should be addressed to the corresponding author.

## Supplementary Material

ciad724_Supplementary_Data

## Notes

***Author contributions.*** D. A. v. S., S. P., D. N., and C. J. S. conceptualized the study. D. N., L. B. S., H. L., D. B., E. V., and C. R. managed patient samples, liaised with clinical teams, and performed laboratory analyses. D. A. v. S., S. P., L. E. N., and J. M.-K. performed in vitro parasite culture and susceptibility assessments. S. P., L. B. S., J. M.-K., H. L., K. B. B., and C. J. S. performed parasite genotyping and sequence analysis. M. B., J. M., L. E. N., A. T., B. C. Y., and P. L. C. managed and assessed patients, provided clinical histories, and secured clinical material for reference laboratory evaluation. C. R., under the direction of P. L. C., managed the reference laboratory. D. A. v. S. and C. J. S., with assistance from B. C. Y. and P. L. C., drafted the manuscript. C. J. S. carried out the statistical analyses. All authors interpreted the results and critically reviewed the manuscript. D. A. v. S. and C. J. S. are the guarantors. The corresponding author attests that all listed authors meet authorship criteria and that no others meeting the criteria have been omitted.

***Data availability.*** Disaggregated in vitro parasite susceptibility datasets are to be made available via the London School of Hygiene and Tropical Medicine (LSHTM) Data Compass Repository (https://datacompass.lshtm.ac.uk/) upon publication. Full genome sequences of each parasite line investigated herein will be deposited in publicly available databases upon completion. All lines are available for use by members of the research community after publication and can be obtained from the authors.

***Disclaimer.*** The funders had no role in considering the study design or in the collection, analysis, interpretation of data, writing of the report, or decision to submit the article for publication. This document expresses opinions of the authors of the paper and may not be understood or quoted as being made on behalf of, or reflecting the position of, the UK Health Security Agency or UK Research and Innovation.

***Financial support.*** This research was supported by the Medical Research Council (research grant MR/T016124/1 awarded to C. J. S.) and the UK Health Security Agency (contract for the UK Malaria Reference Service awarded to LSHTM). B. C. Y. also reports support for this work from University of Oxford (employed as clinical lecturer and honorary researcher) and Oxford University Hospitals (employed as consultant in infection).

## References

[1] Dondorp AM, Nosten F, Yi P, et al Artemisinin resistance in *Plasmodium falciparum* malaria. N Engl J Med 2009; 361:455–67. 19641202 10.1056/NEJMoa0808859 PMC3495232

[2] Noedl H, Se Y, Schaecher K, Smith BL, Socheat D, Fukuda MM. Evidence of artemisinin-resistant malaria in western Cambodia. N Engl J Med 2008; 359:2619–20. 19064625 10.1056/NEJMc0805011

[3] Ariey F, Witkowski B, Amaratunga C, et al A molecular marker of artemisinin-resistant *Plasmodium falciparum* malaria. Nature 2014; 505:50–5. 24352242 10.1038/nature12876 PMC5007947

[4] Menard D, Khim N, Beghain J, et al A worldwide map of *Plasmodium falciparum* K13-propeller polymorphisms. N Engl J Med 2016; 374:2453–64. 27332904 10.1056/NEJMoa1513137 PMC4955562

[5] Balikagala B, Fukuda N, Ikeda M, et al Evidence of artemisinin-resistant malaria in Africa. N Engl J Med 2021; 385:1163–71. 34551228 10.1056/NEJMoa2101746

[6] Uwimana A, Legrand E, Stokes BH, et al Emergence and clonal expansion of in vitro artemisinin-resistant *Plasmodium falciparum kelch13* R561H mutant parasites in Rwanda. Nat Med 2020; 26:1602–8. 32747827 10.1038/s41591-020-1005-2 PMC7541349

[7] Tumwebaze PK, Conrad MD, Okitwi M, et al Decreased susceptibility of *Plasmodium falciparum* to both dihydroartemisinin and lumefantrine in northern Uganda. Nat Commun 2022; 13:6353. 36289202 10.1038/s41467-022-33873-x PMC9605985

[8] Uwimana A, Umulisa N, Venkatesan M, et al Association of *Plasmodium falciparum* kelch13 R561H genotypes with delayed parasite clearance in Rwanda: an open-label, single-arm, multicentre, therapeutic efficacy study. Lancet Infect Dis 2021; 21:1120–8. 33864801 10.1016/S1473-3099(21)00142-0 PMC10202849

[9] Lu F, Culleton R, Zhang M, et al Emergence of indigenous artemisinin-resistant *Plasmodium falciparum* in Africa. N Engl J Med 2017; 376:991–3. 28225668 10.1056/NEJMc1612765

[10] Sutherland CJ, Fifer H, Pearce RJ, et al Novel *pfdhps* haplotypes among imported cases of *Plasmodium falciparum* malaria in the United Kingdom. Antimicrob Agents Chemother 2009; 53:3405–10. 19433569 10.1128/AAC.00024-09 PMC2715629

[11] Sutherland CJ, Lansdell P, Sanders M, et al *pfk13*-independent treatment failure in four imported cases of *Plasmodium falciparum* malaria treated with artemether-lumefantrine in the United Kingdom. Antimicrob Agents Chemother 2017; 61:e02382-16. 28137810 10.1128/AAC.02382-16 PMC5328508

[12] Sonden K, Wyss K, Jovel I, et al High rate of treatment failures in nonimmune travelers treated with artemether-lumefantrine for uncomplicated *Plasmodium falciparum* malaria in Sweden: retrospective comparative analysis of effectiveness and case series. Clin Infect Dis 2017; 64:199–206. 27986683 10.1093/cid/ciw710

[13] van Schalkwyk DA, Burrow R, Henriques G, et al Culture-adapted *Plasmodium falciparum* isolates from UK travellers: in vitro drug sensitivity, clonality and drug resistance markers. Malar J 2013; 12:320. 24028570 10.1186/1475-2875-12-320 PMC3847303

[14] Straimer J, Gnadig NF, Witkowski B, et al Drug resistance. K13-propeller mutations confer artemisinin resistance in *Plasmodium falciparum* clinical isolates. Science 2015; 347:428–31. 25502314 10.1126/science.1260867 PMC4349400

[15] van Schalkwyk DA, Blasco B, Davina Nunez R, et al *Plasmodium knowlesi* exhibits distinct in vitro drug susceptibility profiles from those of *Plasmodium falciparum*. Int J Parasitol Drugs Drug Resist 2019; 9:93–9. 30831468 10.1016/j.ijpddr.2019.02.004 PMC6403410

[16] van Schalkwyk DA, Moon RW, Blasco B, Sutherland CJ. Comparison of the susceptibility of *Plasmodium knowlesi* and *Plasmodium falciparum* to antimalarial agents. J Antimicrob Chemother 2017; 72:3051–8. 28961865 10.1093/jac/dkx279 PMC5890772

[17] Amaratunga C, Neal AT, Fairhurst RM. Flow cytometry-based analysis of artemisinin-resistant *Plasmodium falciparum* in the ring-stage survival assay. Antimicrob Agents Chemother 2014; 58:4938–40. 24867976 10.1128/AAC.02902-14 PMC4136004

[18] Robinson A, Busula AO, Muwanguzi JK, et al Molecular quantification of *Plasmodium* parasite density from the blood retained in used RDTs. Sci Rep 2019; 9:5107. 30911048 10.1038/s41598-019-41438-0 PMC6434039

[19] Sutherland CJ, Haustein T, Gadalla N, Armstrong M, Doherty JF, Chiodini PL. Chloroquine-resistant *Plasmodium falciparum* infections among UK travellers returning with malaria after chloroquine prophylaxis. J Antimicrob Chemother 2007; 59:1197–9. 17475629 10.1093/jac/dkm104

[20] Demas AR, Sharma AI, Wong W, et al Mutations in *Plasmodium falciparum* actin-binding protein coronin confer reduced artemisinin susceptibility. Proc Natl Acad Sci U S A 2018; 115:12799–804. 30420498 10.1073/pnas.1812317115 PMC6294886

[21] Henriques G, Hallett RL, Beshir KB, et al Directional selection at the *pfmdr1*, *pfcrt*, *pfubp1*, and *pfap2mu* loci of *Plasmodium falciparum* in Kenyan children treated with ACT. J Infect Dis 2014; 210:2001–8. 24994911 10.1093/infdis/jiu358 PMC4241946

[22] Witkowski B, Amaratunga C, Khim N, et al Novel phenotypic assays for the detection of artemisinin-resistant *Plasmodium falciparum* malaria in Cambodia: in-vitro and ex-vivo drug-response studies. Lancet Infect Dis 2013; 13:1043–9. 24035558 10.1016/S1473-3099(13)70252-4 PMC5015432

[23] Henrici RC, van Schalkwyk DA, Sutherland CJ. Transient temperature fluctuations severely decrease *P. falciparum* susceptibility to artemisinin in vitro. Int J Parasitol Drugs Drug Resist 2019; 9:23–6. 30599390 10.1016/j.ijpddr.2018.12.003 PMC6312858

[24] Henriques G, van Schalkwyk DA, Burrow R, et al The mu subunit of *Plasmodium falciparum* clathrin-associated adaptor protein 2 modulates in vitro parasite response to artemisinin and quinine. Antimicrob Agents Chemother 2015; 59:2540–7. 25691625 10.1128/AAC.04067-14 PMC4394773

[25] Owoloye A, Olufemi M, Idowu ET, Oyebola KM. Prevalence of potential mediators of artemisinin resistance in African isolates of *Plasmodium falciparum*. Malar J 2021; 20:451. 34856982 10.1186/s12936-021-03987-6 PMC8638531

[26] Henrici RC, van Schalkwyk DA, Sutherland CJ. Modification of *pfap2mu* and *pfubp1* markedly reduces ring-stage susceptibility of *Plasmodium falciparum* to artemisinin in vitro. Antimicrob Agents Chemother 2019; 64:e01542-19. 31636063 10.1128/AAC.01542-19 PMC7187599

[27] Gadalla NB, Adam I, Elzaki SE, et al Increased *pfmdr1* copy number and sequence polymorphisms in *Plasmodium falciparum* isolates from Sudanese malaria patients treated with artemether-lumefantrine. Antimicrob Agents Chemother 2011; 55:5408–11. 21896916 10.1128/AAC.05102-11 PMC3195064

[28] Humphreys GS, Merinopoulos I, Ahmed J, et al Amodiaquine and artemether-lumefantrine select distinct alleles of the *Plasmodium falciparum mdr1* gene in Tanzanian children treated for uncomplicated malaria. Antimicrob Agents Chemother 2007; 51:991–7. 17194834 10.1128/AAC.00875-06 PMC1803116

[29] Sisowath C, Petersen I, Veiga MI, et al In vivo selection of *Plasmodium falciparum* parasites carrying the chloroquine-susceptible *pfcrt* K76 allele after treatment with artemether-lumefantrine in Africa. J Infect Dis 2009; 199:750–7. 19210165 10.1086/596738 PMC2718568

[30] Sisowath C, Stromberg J, Martensson A, et al In vivo selection of *Plasmodium falciparum pfmdr1* 86N coding alleles by artemether-lumefantrine (Coartem). J Infect Dis 2005; 191:1014–7. 15717281 10.1086/427997

[31] Tumwebaze PK, Katairo T, Okitwi M, et al Drug susceptibility of *Plasmodium falciparum* in eastern Uganda: a longitudinal phenotypic and genotypic study. Lancet Microbe 2021; 2:e441–9. 34553183 10.1016/s2666-5247(21)00085-9 PMC8454895

[32] Conrad MD, Asua V, Garg S, et al Evolution of partial resistance to artemisinins in malaria parasites in Uganda. N Engl J Med 2023; 389:722–32. 37611122 10.1056/NEJMoa2211803 PMC10513755

[33] Mihreteab S, Platon L, Berhane A, et al Increasing prevalence of artemisinin-resistant HRP2-negative malaria in Eritrea. N Engl J Med 2023; 389:1191–202. 37754284 10.1056/NEJMoa2210956 PMC10539021

[34] Schallig HD, Tinto H, Sawa P, et al Randomised controlled trial of two sequential artemisinin-based combination therapy regimens to treat uncomplicated falciparum malaria in African children: a protocol to investigate safety, efficacy and adherence. BMJ Glob Health 2017; 2:e000371. 10.1136/bmjgh-2017-000371 PMC5656137 29082016

