# Supplementary material for: Treatment Failure in a UK Malaria Patient Harboring Genetically Variant Plasmodium falciparum From Uganda With Reduced In Vitro Susceptibility to Artemisinin and Lumefantrine
Source: Clin Infect Dis. 2023 Nov 29;78(2):445–52. doi: 10.1093/cid/ciad724 (PMC10874266; doi:10.1093/cid/ciad724)
Supplement: ciad724_Supplementary_Data [file ciad724_supplementary_data.docx]

S160N

PF3D7 MIDALYIFFINGQLLIQRNYRDTTKRTDLTQYINKYIKTKRFYENPIVEINNVFFINVNINEIVITVLTRSNSNICLIFNFIYKFIEILKYFFNNELSGINIVNNFVLIYEICDEIIDYGYPQTLEVNILKNSLLNKVKYYSKTSRYFQKISNELLNVNSVIED

HL1601 MIDALYIFFINGQLLIQRNYRDTTKRTDLTQYINKYIKTKRFYENPIVEINNVFFINVNINEIVITVLTRSNSNICLIFNFIYKFIEILKYFFNNELSGINIVNNFVLIYEICDEIIDYGYPQTLEVNILKNSLLNKVKYYSKTSRYFQKISNELLNVNSVIED

HL2206 MIDALYIFFINGQLLIQRNYRDTTKRTDLTQYINKYIKTKRFYENPIVEINNVFFINVNINEIVITVLTRSNSNICLIFNFIYKFIEILKYFFNNELSGINIVNNFVLIYEICDEIIDYGYPQTLEVNILKNSLLNKVKYYSKTSRYFQKISNELLNVNSVIED

Hl2208 MIDALYIFFINGQLLIQRNYRDTTKRTDLTQYINKYIKTKRFYENPIVEINNVFFINVNINEIVITVLTRSNSNICLIFNFIYKFIEILKYFFNNELSGINIVNNFVLIYEICDEIIDYGYPQTLEVNILKNSLLNKVKYYSKTSRYFQKISNELLNVNNVIED

**HL2210** MIDALYIFFINGQLLIQRNYRDTTKRTDLTQYINKYIKTKRFYENPIVEINNVFFINVNINEIVITVLTRSNSNICLIFNFIYKFIEILKYFFNNELSGINIVNNFVLIYEICDEIIDYGYPQTLEVNILKNSLLNKVKYYSKTSRYFQKISNELLNVNSVIED

HL2211 MIDALYIFFINGQLLIQRNYRDTTKRTDLTQYINKYIKTKRFYENPIVEINNVFFINVNINEIVITVLTRSNSNICLIFNFIYKFIEILKYFFNNELSGINIVNNFVLIYEICDEIIDYGYPQTLEVNILKNSLLNKVKYYSKTSRYFQKISNELLNVNSVIED

HL2212 MIDALYIFFINGQLLIQRNYRDTTKRTDLTQYINKYIKTKRFYENPIVEINNVFFINVNINEIVITVLTRSNSNICLIFNFIYKFIEILKYFFNNELSGINIVNNFVLIYEICDEIIDYGYPQTLEVNILKNSLLNKVKYYSKTSRYFQKISNELLNVNSVIED

HL2213 MIDALYIFFINGQLLIQRNYRDTTKRTDLTQYINKYIKTKRFYENPIVEINNVFFINVNINEIVITVLTRSNSNICLIFNFIYKFIEILKYFFNNELSGINIVNNFVLIYEICDEIIDYGYPQTLEVNILKNSLLNKVKYYSKTSRYFQKISNELLNVNSVIED

HL2214 MIDALYIFFINGQLLIQRNYRDTTKRTDLTQYINKYIKTKRFYENPIVEINNVFFINVNINEIVITVLTRSNSNICLIFNFIYKFIEILKYFFNNELSGINIVNNFVLIYEICDEIIDYGYPQTLEVNILKNSLLNKVKYYSKTSRYFQKISNELLNVNSVIED

***************************************************************************************************************************************************************.****

N-tract #2: 5N-TS-4N; 6N-TS-4N; 5N-TS-5N

N-tract #1: 7N-K, 7N-2K

PF3D7 IVHDPHIHNRTNKSNNKIRDFYNTKSVKNKNTYDLNETNKLKYIGKETLNRIKNKIINNNNNNN-KTANHFNYITGNCTWRNNNIYYKKNEIYIDILEILNVTINSNNLIYAHINGKVTLKCHLSGMPLCELSTNNKINLLKNILAGSNTSNNNNN-TSNNNN-K

HL1601 IVHDPHIHNRTNKSNNKIRDFYNTKSVKNKNTYDLNETNKLKYIGKETLNRIKNKIINNNNNNN-KTANHFNYITGNCTWRNNNIYYKKNEIYIDILEILNVTINSNNLIYAHINGKVTLKCHLSGMPLCELSTNNKINLLKNILAGSNTSNNNNN-TSNNNN-K

HL2206 IVHDPHIHNRTNKSNNKIRDFYNTKSVKNKNTYDLNETNKLKYIGKETLNRIKNKIINNNNNNN-KTANHFNYITGNCTWRNNNIYYKKNEIYIDILEILNVTINSNNLIYAHINGKVTLKCHLSGMPLCELSTNNKINLLKNILAGSNTSNNNNN-TSNNNN-K

Hl2208 IVHDPHIHNRTNKSNNKIRDFYNTKSVKNKNTYDLNETNKLKYIGKETLNRIKNKIINNNNNNN-KTANHFNYITGNCTWRNNNIYYKKNEIYIDILEILNVTINSNNLIYAHINGKVTLKCHLSGMPLCELSTNNKINLLKNILAGSNTSNNNNN-TSNNNNNK

**HL2210** IVHDPHIHNRTNKSNNKIRDFYNTKSVKNKNTYDLNETNKLKYIGKETLNRIKNKIINNNNNNN-KTANHFNYITGNCTWRNNNIYYKKNEIYIDILEILNVTINSNNLIYAHINGKVTLKCHLSGMPLCELSTNNKINLLKNILAGSNTSNNNNN-TSNNNN-K

HL2211 IVHDPHIHNRTNKSNNKIRDFYNTKSVKNKNTYDLNETNKLKYIGKETLNRIKNKIINNNNNNN-KTANHFNYITGNCTWRNNNIYYKKNEIYIDILEILNVTINSNNLIYAHINGKVTLKCHLSGMPLCELSTNNKINLLKNILAGSNTSNNNNNNTSNNNN-K

HL2212 IVHDPHIHNRTNKSNNKIRDFYNTKSVKNKNTYDLNETNKLKYIGKETLNRIKNKIINNNNNNN-KTANHFNYITGNCTWRNNNIYYKKNEIYIDILEILNVTINSNNLIYAHINGKVTLKCHLSGMPLCELSTNNKINLLKNILAGSNTSNNNNN-TSNNNN-K

HL2213 IVHDPHIHNRTNKSNNKIRDFYNTKSVKNKNTYDLNETNKLKYIGKETLNRIKNKIINNNNNNNKKTANHFNYITGNCTWRNNNIYYKKNEIYIDILEILNVTINSNNLIYAHINGKVTLKCHLSGMPLCELSTNNKINLLKNILAGSNTSNNNNN-TSNNNN-K

HL2214 IVHDPHIHNRTNKSNNKIRDFYNTKSVKNKNTYDLNETNKLKYIGKETLNRIKNKIINNNNNNN-KTANHFNYITGNCTWRNNNIYYKKNEIYIDILEILNVTINSNNLIYAHINGKVTLKCHLSGMPLCELSTNNKINLLKNILAGSNTSNNNNN-TSNNNN-K

**************************************************************** ******************************************************************************************* ****** *

F437LN

PF3D7 TNQGNALRGSCGSNSLVNNKVMQNNLKKKYTLDEKDNEEIIIDNCIFHHCVTLSKYENNKVITFTPPDGTFELMKYTITKNIQIPFHILAIYNPILEYSKNVEKKFSLKKLTTNNKSIYGEYKNTNKYEYSVTIKSNYKGNMHASDVLIKIPIYKFSENVQVKY

HL1601 TNQGNALRGSCGSNSLVNNKVMQNNLKKKYTLDEKDNEEIIIDNCIFHHCVTLSKYENNKVITFTPPDGTFELMKYTITKNIQIPFHILAIYNPILEYSKNVEKKFSLKKLTTNNKSIYGEYKNTNKYEYSVTIKSNYKGNMHASDVLIKIPIYKFSENVQVKY

HL2206 TNQGNALRGSCGSNSLVNNKVMQNNLKKKYTLDEKDNEEIIIDNCIFHHCVTLSKYENNKVITFTPPDGTFELMKYTITKNIQIPFHILAIYNPILEYSKNVEKKFSLKKLTTNNKSIYGEYKNTNKYEYSVTIKSNYKGNMHASDVLIKIPIYKFSENVQVKY

Hl2208 TNQGNALRGSCGSNSLVNNKVMQNNLKKKYTLDEKDNEEIIIDNCIFHHCVTLSKYENNKVITFTPPDGTFELMKYTITKNIQIPFHILAIYNPILEYSKNVEKKFSLKKLTTNNKSIYGEYKNTNKYEYSVTIKSNYKGNMHASDVLIKIPIYKFSENVQVKY

**HL2210** TNQGNALRGSCGSNSLVNNKVMQNNLKKKYTLDEKDNEEIIIDNCIFHHCVTLSKYENNKVITFTPPDGTFELMKYTITKNIQIPFHILAIYNPILEYSKNVEKKFSLKKLTTNNKSIYGEYKNTNKYEYSVTIKSNYKGNMHASDVLIKIPIYKFSENVQVKY

HL2211 TNQGNALRGSCGSNSLVNNKVMQNNLKKKYTLDEKDNEEIIIDNCIFHHCVTLSKYENNKVITFTPPDGTFELMKYTITKNIQIPFHILAIYNPILEYSKNVEKKFSLKKLTTNNKSIYGEYKNTNKYEYSVTIKSNYKGNMHASDVLIKIPIYKFSENVQVKY

HL2212 TNQGNALRGSCGSNSLVNNKVMQNNLKKKYTLDEKDNEEIIIDNCIFHHCVTLSKYENNKVITFTPPDGTFELMKYTITKNIQIPFHILAIYNPILEYSKNVEKKFSLKKLTTNNKSIYGEYKNTNKYEYSVTIKSNYKGNMHASDVLIKIPIYKFSENVQVKY

HL2213 TNQGNALRGSCGSNSLVNNKVMQNNLKKKYTLDEKDNEEIIIDNCIFHHCVTLSKYENNKVITFTPPDGTFELMKYTITKNIQIPFHILAIYNPILEYSKNVEKKLSLKKLTTNNKSIYGEYKNTNKYEYSVTIKSNYKGNMHASDVLIKIPIYKFSENVQVKY

HL2214 TNQGNALRGSCGSNSLVNNKVMQNNLKKKYTLDEKDNEEIIIDNCIFHHCVTLSKYENNKVITFTPPDGTFELMKYTITKNIQIPFHILAIYNPILEYSKNVEKKFSLKKLTTNNKSIYGEYKNTNKYEYSVTIKSNYKGNMHASDVLIKIPIYKFSENVQVKY

*********************************************************************************************************:**********************************************************

PF3D7 KSIGKTEFNNIDSLVIWRIKKFLSSSEHNIKIHLTLENHNQIYSNMNNTQKVDDLSKVVLQVHKIKNMNTVKFLNTYKMPITLSFKIPMFTSSGMYIRYLKVFEKSNYKIIKWIKY

HL1601 KSIGKTEFNNIDSLVIWRIKKFLSSSEHNIKIHLTLENHNQIYSNMNNTQKVDDLSKVVLQVHKIKNMNTVKFLNTYKMPITLSFKIPMFTSSGMYIRYLKVFEKSNYKIIKWIKY

HL2206 KSIGKTEFNNIDSLVIWRIKKFLSSSEHNIKIHLTLENHNQIYSNMNNTQKVDDLSKVVLQVHKIKNMNTVKFLNTYKMPITLSFKIPMFTSSGMYIRYLKVFEKSNYKIIKWIKY

Hl2208 KSIGKTEFNNIDSLVIWRIKKFLSSSEHNIKIHLTLENHNQIYSNMNNTQKVDDLSKVVLQVHKIKNMNTVKFLNTYKMPITLSFKIPMFTSSGMYIRYLKVFEKSNYKIIKWIKY

**HL2210** KSIGKTEFNNIDSLVIWRIKKFLSSSEHNIKIHLTLENHNQIYSNMNNTQKVDDLSKVVLQVHKIKNMNTVKFLNTYKMPITLSFKIPMFTSSGMYIRYLKVFEKSNYKIIKWIKY

HL2211 KSIGKTEFNNIDSLVIWRIKKFLSSSEHNIKIHLTLENHNQIYSNMNNTQKVDDLSKVVLQVHKIKNMNTVKFLNTYKMPITLSFKIPMFTSSGMYIRYLKVFEKSNYKIIKWIKY

HL2212 KSIGKTEFNNIDSLVIWRIKKFLSSSEHNIKIHLTLENHNQIYSNMNNTQKVDDLSKVVLQVHKIKNMNTVKFLNTYKMPITLSFKIPMFTSSGMYIRYLKVFEKSNYKIIKWIKY

HL2213 KSIGKTEFNNIDSLVIWRIKKFLSSSEHNIKIHLTLENHNQIYSNMNNTQKVDDLSKVVLQVHKIKNMNTVKFLNTYKMPITLSFKIPMFTSSGMYIRYLKVFEKSNYKIIKWIKY

HL2214 KSIGKTEFNNIDSLVIWRIKKFLSSSEHNIKIHLTLENHNQIYSNMNNTQKVDDLSKVVLQVHKIKNMNTVKFLNTYKMPITLSFKIPMFTSSGMYIRYLKVFEKSNYKIIKWIKY

********************************************************************************************************************

**Supplementary Figure 1. Alignment of UBP-1 amino acid sequences from parasite lines evaluated in this study.** Clustal omega alignment of full-length amino acid sequences encoded by *pfap2mu* genes from the index malaria patient and 6 comparator lines from Uganda. Non-synonymous SNP and asparagine repeat regions are highlighted, with variants listed. HL2213 harboured at least two alleles of *ap2mu*; a composite sequence is shown capturing non-reference features which may not have been present in a single haplotype. The F437L and S160N mutations have been previously described in Kenya at low frequency; their impact on drug susceptibility is equivocal and may depend on genetic variation at other loci. Both variants have been described in samples collected in the 1990s, pre-dating the introduction of ACT in Africa [22,24,26].

Three amino acid repeats:

Ref QDWSSYTKDKENKLNMDDDINMNKGNDQDVNRTYKNEKNKEEDKYGKNEKNEKYDKYD------KYEKYE---KYDKYKKDNKNQHDDPLYDNINKNYDNDNKG

HL1601 QDWSSYTKDKENKLNMDDDINMNKGNDQDVNRTYKNEKNKEEDKYGKNEKNEKYD------KYEKYEKYE---KYDKYKKDNKNQHDDPLYDNINKNYDNDNKG

HL2206 QDWSSYTKDKENKLNMDDDINMNKGNDQDVNRTYKNEKNKEEDKYGKNEKNEKYDKYD------KYEKYE---KYDKYKKDNKNQHDDPLYDNINKNYDNDNKG

HL2208 QDWSSYTKDKENKLNMDDDINMNKGNDQDVNRTYKNEKNKEEDKYGKNEKNEKYDKYD------KYEKYE---KYDKYKKDNKNQHDDPLYDNINKNYDNDNKG

**HL2210** QDWSSYTKDKENKLNMDDDINMNKGNDQDVNRTYKNEKNKEEDKYGKNEKNEKYDKYDKYD------KYE**KCE**KYDKYKKDNKNQHDDPLYDNINKNYDNDNKG

HL2212 QDWSSYTKDKENKLNMDDDINMNKGNDQDVNRTYKNEKNKEEDKYGKNEKNEKYDKYD------KYEKYE---KYDKYKKDNKNQHDDPLYDNINKNYDNDNKG

HL2213 QDWSSYTKDKENKLNMDDDINMNKGNDQDVNRTYKNEKNKEEDKYGKNEKNEKYDKYD------KYEKYE---KYDKYKKDNKNQHDDPLYDNINKNYDNDNKG

HL2214 QDWSSYTKDKENKLNMDDDINMNKGNDQDVNRTYKNEKNKEEDKYGKNEKNEKYDKYD---------KYE---KYDKYKKDNKNQHDDPLYDNINKNYDNDNKG

Nine amino acid repeats:

Ref  NHVNRMDGVNHVNRMDGVNHVNRMDGVNRVN---------RMNHANRVSRMNHANRVSRMNHANRVSRMNHANRVSPNNIEDIRMGGVKIKKYLMLPINKFTFENM

HL1601  NHVNRMDGVNHVNRMDGVNHVNRMDGVNRVN------------------RMNHANRVSRMNHANRVSRMNHANRVSPNNIEDIRMGGVKIKKYLMLPINKFTFENM

HL2206  NHVNRMDGVNHVNRMDGVNHVNRMDGVNRVN------------------RMNHANRVSRMNHANRVSRMNHANRVSPNNIEDIRMGGVKIKKYLMLPINKFTFENM

HL2208 NHVNRMDGVNHVNRMDGVNHVNRMDGVNRVN------------------RMNHANRVS**L**MNHANRVSRMNHANRVSPNNIEDIRMGGVKIKKYLMLPINKFTFENM

**HL2210**  NHVNRMDGVNHVNRMDGVNHVNRMDGVNRVN---------RMNHANRVSRMNHANRVSRMNHANRVSRMNHANRVSPNNIEDIRMGGVKIKKYLMLPINKFTFENM

HL2212  NHVNRMDGVNHVN------------------RMDGVNRVS------------------RMNHANRVSRMNHANRVSPNNIEDIRMGGVKIKKYLMLPINKFTFENM

HL2213  NHVNRMDGVNHVNRMDGVNHVNRMDGVNRVN------------------RMNHANRVSRMNHANRVSRMNHANRVSPNNIEDIRMGGVKIKKYLMLPINKFTFENM

**Supplementary Figure 2. Alignment of UBP-1 amino acid repeat sequences from parasite lines evaluated in this study.** Clustal omega alignment of amino acids encoded by codons 1468-1562 (above) and 3064-3160 (below) of the *pfubp1* reference sequence (Pf3D7_0104300) are shown aligned with the seven cultured isolates. The index case is indicated at left by highlighting. The three amino acid repeat region was first identified by Borrman et al [PLoS One **2011**; 6(11): e26005]. Sequence diversity and repeat number variation in the six amino acid repeat region has not been previously described in detail. Both regions are in domains without substantial homology to proteins encoded in other eukaryotic genomes and are likely to be *Plasmodium-*specific. Full sequence data was not available for HL2214.
